# SNX16 negatively regulates the migration and tumorigenesis of MCF-7 cells

**DOI:** 10.1186/2045-9769-2-3

**Published:** 2013-04-30

**Authors:** Leilei Zhang, Dajiang Qin, Chunfang Hao, Xiaodong Shu, Duanqing Pei

**Affiliations:** Key Laboratory of Regenerative Biology, Chinese Academy of Sciences, and Guangdong Provincial Key Laboratory of Stem Cells and Regenerative Medicine, South China Institute for Stem Cell Biology and Regenerative Medicine, Guangzhou Institutes of Biomedicine and Health, Guangzhou, 510530 China

## Abstract

**Background:**

Sorting nexins are a large family of proteins that are associated with various components of the endosome system and they play many roles in processes such as endocytosis, intracellular protein trafficking and cell signaling. The subcellular distribution patterns of many of them remain controversial and their *in vivo* functions have not been characterized yet.

**Results:**

We investigated the subcellular distribution and function of SNX16 in this study. SNX16 is detected on Rab5-positive endosomes localized adjacent to focal adhesions at cell cortex. Inhibition of SNX23, polymerization of microtubule filaments as well as the PI3-kinase all disrupt the cell cortex distribution of SNX16. Ectopic expression of SNX16 reduces the migration and the tumor formation activity of MCF-7 cells.

**Conclusion:**

Our results indicate that, in addition to the PI3P, there is a SNX23- and microtubule-dependent cargo transport pathway required for the proper subcellular distribution of SNX16. SNX16 plays a negative regulatory role during cell migration and tumorigenesis.

## Background

Sorting nexin family proteins (SNXs) all contain a Phox-homology (PX) domain which binds to certain phosphoinositides and targets the host protein to organelles rich in those lipids [1, 2]. SNX genes are present in all eukaryotes from yeast to mammals and 33 SNX family members have been identified from the mouse and human genome. Twelve members of the mammalian SNX family (SNX1, 2, 4–9, 18, 30, 32 and 33) contain a BAR (Bin, amphiphysin, Rvs) domain next to the PX domain and they are grouped into the PX-BAR subfamily of SNXs. The BAR domain can sense membrane curvature and many of the PX-BAR subfamily SNX members are involved in the retromer-dependent vesicular trafficking [3–5]. The classic mammalian retromer consists of a cargo-selective adaptor (Vps26-29-35) and a membrane-bound heterodimer of SNX1/2 and SNX5/6. It regulates the retrograde trafficking of cargos such as the cation-independent mannose-6-phosphate receptor (CI-MPR) from endosomes to the Golgi apparatus. Recently, SNX3 which is a PX-domain-only SNX family member has been demonstrated to play an essential role in a novel type of retromer-dependent trafficking of Wntless [6, 7]. SNX10 is another PX-domain-only SNX protein which is able to regulate the subcellular distribution of vacuolar-type H^+^-ATPase (V-ATPase) [8] and it has recently been implicated in hereditary osteopetrosis in human [9–12].

Many SNX family members contain protein domains other than the PX or BAR domain. For example, SNX17 contains a FERM (4.1, Ezrin, Radixin, Moesin) domain [13, 14] and it has been implicated in the intracellular sorting and trafficking of membrane proteins including P-selectin [15], low density lipoprotein receptor (LDLR) [16], LDLR related protein (LRP) [17, 18], integrin [19, 20], Jag1 [21], etc.. SNX27 contains a PDZ (postsynaptic density protein-95, Discs-large, Zona-occludens-1) domain and a Ras-association domain in addition to the PX domain. It is involved in the regulation of the G protein-gated inwardly rectifying potassium (GIRK) channel [22, 23], the β_2_-adrenoreceptor [24, 25], the 5-hydroxytryptamine type 4 receptor [26], the N-methyl-D-aspartate receptor 2C [27] as well as the glutamate receptors [28]. SNX23 (also known as KIF16B) contains a kinesin motor domain and it can regulate the microtubule-dependent Golgi-to-endosome transport of the fibroblast growth factor receptor (FGFR) [29] or the cell peripheral transport of early endosomes [30]. SNX16 is another unique member of the SNX family in that it contains a coiled-coil (CC) domain next to the C-end of the PX domain. The PX domain binds to the phosphatidylinositol 3-phosphate (PI3P) and targets SNX16 to the early and late endosomes [31]. More detailed analysis reveals that SNX16 is distributed to the Rab7-positive late endosomes but not the phospholipid lysobisphosphatidic acid (LBPA)-positive late endosome/multivesicular endosomes [32]. In COS-7 cells, SNX16 co-localizes with the transferrin receptor (TFR) and is able to enhance the EGF-induced degradation of EGF receptor [33]. In drosophila cells, SNX16 is detected at early endosomes and it can activate the BMP signaling which is required for synaptic growth [34].

We report here that SNX16 is often detected on vesicles at cell cortex. These vesicles are Rab5-positive and they are distributed close to the focal adhesions. The activity of SNX23, the microtubule filaments as well as the PI3-kinase are all required for the cell cortex distribution of SNX16. Over-expression of SNX16 reduces the migration of cells while knockdown of SNX16 has the opposite effect. Furthermore, ectopic expression of SNX16 is able to reduce the *in vivo* tumorigenic activity of a breast cancer cell line in the mouse model.

## Results

### Cell cortex distribution of SNX16 *in vitro* and *in vivo*

**Figure 1:**
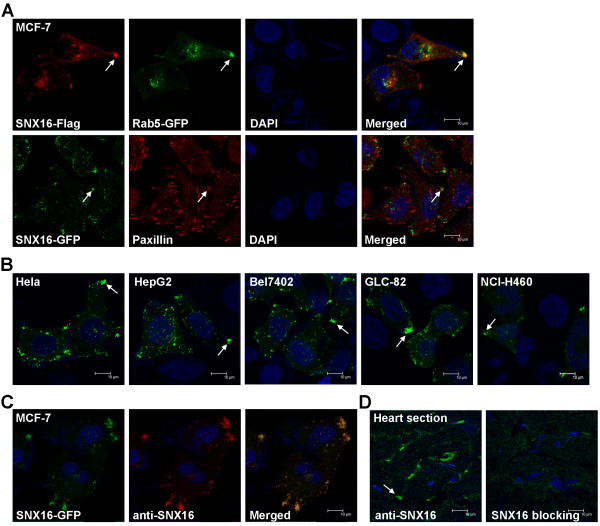
**Subcellular distribution of SNX16.** (**A**) The subcellular distribution of tagged SNX16 in MCF-7 cells. Cells were transfected with the indicated constructs and immunofluoresence staining was performed 48 hrs post transfection. Rab5 is an early endosome marker and it co-localizes with SNX16 at cell cortex (indicated by an arrow). The endogenous Paxillin is detected using a specific antibody and used to indicate the position of focal adhesions. (**B**) The cell cortex distribution of SNX16 is detectable in a variety of cell lines. (**C**) A home-made polyclonal antibody to human SNX16 can detect the ectopically expressed SNX16. (**D**) Immunofluoresence staining of endogenous SNX16 on frozen sections prepared from adult mouse heart. Pre-incubation of the sample with soluble SNX16 protein blocks the staining.

SNX16 has been detected at various endosome compartments including early endosomes, late endosomes/lysosomes or recycling endosomes; however, the exact subcellular distribution of SNX16 appears to be cell line dependent [31–34]. We initially investigated the distribution of ectopic SNX16 (Flag- or GFP-tagged) in MCF-7 which is a commonly used cell line derived from human breast cancer. We found that, in addition to the perinuclear region of cytoplasm, SNX16 vesicles are accumulated at certain cell cortex (indicated by arrows in Figure 1A). These vesicles are Rab5-positive so they are likely to be early endosomes. This distinct distribution pattern of SNX16 prompted us to investigate whether or not it is related to the focal adhesions, where a cell is linked to the extracellular matrix. Paxillin is a focal adhesion-associated adaptor protein and it is used to indicate the position of focal adhesions. We found that the cell cortex fraction of SNX16 is always adjacent to the Paxillin staining signals but they usually do not co-localize with each other. So we conclude that SNX16 vesicles are accumulated near certain focal adhesions at the peripheral cytoplasm in MCF-7 cells.

We then investigated whether or not the cell cortex distribution is a general feature for SNX16. We transfected SNX16-GFP into various cell lines and determined the subcellular distribution of SNX16 in these cells. We found that the cell cortex localization of SNX16 is clearly detected in all cell lines examined, which include a cervical cancer cell line (Hela), liver cancer cell lines (HepG2 and Bel7402) and lung cancer cell lines (GLC-82 and NCI-H460) (Figure 1B). We then investigated whether the cell cortex distribution of SNX16 can be found *in vivo*. We first developed a polyclonal antibody against SNX16 and this antibody successfully detects the ectopically expressed SNX16-GFP in MCF-7 cells (Figure 1C). SNX16 is enriched in brain and muscles in mouse [31], so we tested whether SNX16 is distributed to the cell cortex in these tissues. We performed immunofluorensence staining on mouse heart frozen sections using our home-made antibody. Cell cortex staining of SNX16 is detected at mouse heart sections but not the same sample pre-blocked with the purified SNX16 soluble protein (Figure 1D). This result suggests that the staining is specific and we conclude that a fraction of SNX16 is present at cell cortex both *in vitro* and *in vivo*.

### Signals required for the cell cortex distribution of SNX16

**Figure 2:**
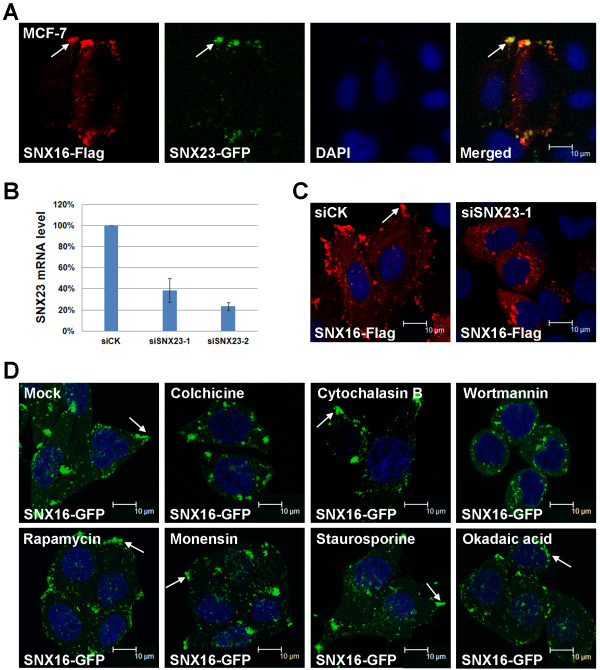
**SNX23 and PI3-kinase are required for the cell cortex distribution of SNX16.** (**A**) SNX16 co-localizes with SNX23 to cell cortex. MCF-7 cells were transfected with the Flag-tagged SNX16 and the GFP-tagged SNX23 and the subcellular distribution of them determined as described above. (**B**) Efficiency of siRNAs to SNX23 as determined by real-time RT-PCR. (**C**) Down-regulation of SNX23 by siRNA disrupts the cell cortex distribution of SNX16. (**D**) The effects of small chemical inhibitors on the subcellular distribution of SNX16. Cells expressing SNX16-GFP were treated with the indicated inhibitors and the subcellular distribution of SNX16 determined as described above. Inhibition the polymerization of microtubules by colchicine or inhibition the activity of PI3-kinase by wortmannin abolishes the cell cortex distribution of SNX16. Inhibition of actin filaments (cytochalasin B) or mTOR (rapamycin) does not affect the cell cortex distribution of SNX16. Treatment of cells with monensin, staurosporine or okadaic acid has no effect on the cell cortex distribution of SNX16.

SNX23/KIF16B is a kinesin family protein that can regulate the microtubule-based peripheral transport of early endosomes. It is reported to co-localize with early endosome marker EEA1 at the cell cortex in Hela cells [30]. This distribution pattern of SNX23 is similar to what we observed for SNX16 here, so we compared the subcellular distribution patterns of SNX16 and SNX23. We co-transfected SNX16 and 23 into the MCF-7 cells and found that they co-localize with each other at cell cortex (Figure 2A). Since SNX23 is a motor protein that can regulate the cell peripheral transport of early endosomes, we determined whether the SNX23 transport pathway is required for the cell cortex distribution of SNX16. We knocked-down SNX23 by siRNAs then determined the subcellular distribution pattern of SNX16. Our siRNAs effectively down-regulate the mRNA level of SNX23 (Figure 2B) and we found that down-regulation of SNX23 abolishes the peripheral distribution of SNX16. In fact, the majority of SNX16 vesicles are now detected at the perinuclear regions (Figure 2C, similar result was observed for siSNX23-2). The microtubule filaments are required for the SNX23-mediated cargo transport [30], so we investigated whether the microtubules are involved in the trafficking of SNX16 vesicles. Pretreatment of MCF-7 cells with colchicine, an inhibitor of microtubule polymerization, disrupts the cortex localization of SNX16 vesicles. On the other hand, inhibition of the actin filaments by cytochalasin B does not affect the cell cortex distribution of SNX16 (Figure 2D). So, the SNX23- and microtubule-dependent transport route is required for the cell cortex distribution of SNX16 vesicles.

The PX domain of SNX16 can bind to PI3P thus the PI3-kinase pathway is able to regulate the early endosome localization of SNX16 [31, 33]. We analyzed whether the PI3-kinase pathway is involved in the cell cortex distribution of SNX16 as well. We found that the inhibition of PI3-kinase by small chemical wortmannin abolishes the cell cortex localization of SNX16 vesicles (Figure 2D). On the other hand, inhibition of mTOR which is a PI3K-related kinase by rapamycin does not induce similar effect. Treatment of cells with an inhibitor of intracellular protein trafficking (monensin), a general inhibitor of protein kinases (staurosporine) or an inhibitor of serine/threonine protein phosphatases (okadaic acid), does not disrupt the cell cortex distribution of SNX16 (Figure 2D). Together, these results suggest that both the PI3-kinase pathway and the SNX23/microtubule system are involved in the establishment or maintenance of SNX16 vesicles at cell cortex.

### SNX16 regulates cell migration but not growth

**Figure 3:**
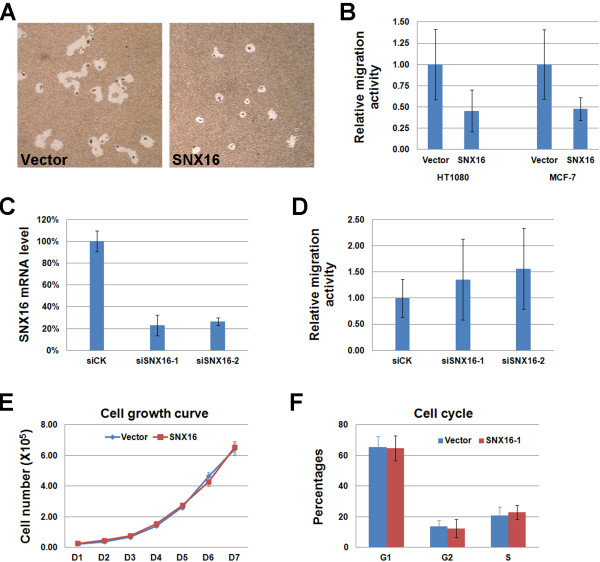
**SNX16 regulates the migration but not proliferation of cells.** (**A**) Stable cell lines expressing SNX16 or an empty vector were established in the HT1080 or MCF-7 cells and the migration activities of these cells were evaluated by the cell migration assay. A typical result of the assay is shown here. (**B**) Statistical analysis of (**A**). Ectopic expression of SNX16 reduces the migration of both HT1080 and MCF-7 cells. (**C**) Both siRNAs to SNX16 efficiently reduce the mRNA level of SNX16 in MCF-7 cells as determined by real-time RT-PCR. (**D**) Down-regulation of SNX16 by either siRNA enhances the migration of MCF-7 cells. (**E, F**) Ectopic expression of SNX16 does not change the growth curve (**E**) or cell cycle profile (**F**) of MCF-7 cells. Data represent mean ± SD in all cases.

Previous studies have implicated SNX16 in the signaling pathways such as EGF, BMP and Wnt pathways [33, 34]. These pathways have diverse functions in regulating processes such as cell survival, proliferation or migration. Our observation that SNX16 is present close to focal adhesions further suggests that it might be involved in cell migration. In order to test this possibility, we first established cell lines stably expressing SNX16 in MCF-7 and HT1080 cells. We compared the migration activity of SNX16-expressing cells to the empty vector infected cells using the Cell Motility HCS Reagent Kit. We found that ectopic expression of SNX16 reduces the migration of both cells to less than half of the control levels (Figure 3A and B, P=2.4×10^-7^ for MCF-7, P=3.4×10^-19^ for HT1080). We then performed loss-of-function assay on SNX16 and found that the siRNA mediated knockdown of SNX16 enhances the migration of MCF-7 cells (Figure 3C and D, P=0.04 for siSNX16-1 and 0.02 for siSNX16-2 when compared to siCK). We compared the growth curve and cell cycle profile between the vector and SNX16 expressing MCF-7 stable cell lines and found that they are not affected by SNX16 over expression (Figure 3E and F). Together, these results suggest that SNX16 is involved in cell migration but not growth.

### SNX16 regulates tumorigenesis of MCF-7 cells

**Figure 4:**
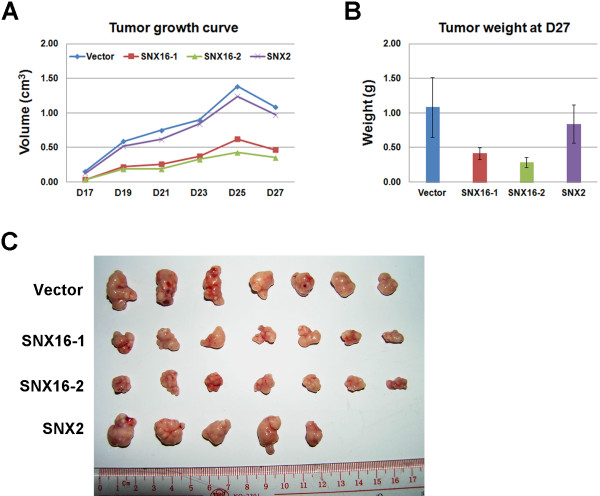
**SNX16 negatively regulates tumorigenesis of MCF-7 cells** ***in vivo*** **.** (**A**) Stable MCF-7 cell lines expressing SNX16, SNX2 or the empty vector were injected subcutaneously into the SCID mice and the sizes of tumors formed at the indicated time (day) were determined. (**B, C**) Tumors were dissected and weighted 27 days post inoculation. Over-expression of SNX16 but not SNX2 reduces the tumorigenic activity of MCF-7 cells. Data represent mean ± SD from 7 mice (for Vector or SNX16) or 5 mice (for SNX2).

MCF-7 is a breast cancer derived cell line that can induce tumor formation when injected subcutaneously into the SCID mice. We investigated whether or not the ectopic expression of SNX16 has an effect on the tumorigenic activity of this cell line. Stable MCF-7 cell lines expressing the empty vector or SNX2 are used as the control. We injected these cells into the SCID mice, monitored the sizes of the tumors and finally determined the weights of tumors 27 days post inoculation after the dissection of tumors. We found that the ectopic expression of SNX16 but not SNX2 significantly reduces the tumor formation activity of MCF-7 cells (Figure 4, P=0.001, 0.0002 and 0.27 for SNX16-1, SNX16-2 and SNX2 respectively). Together, our results suggest that SNX16 is a negative regulator of cell migration and tumorigenesis *in vivo*.

## Discussion

SNX16 contains a PX domain and a C-terminal coiled-coil domain, which is unique among SNX family members. Previous biochemical studies demonstrate that the PX domain of SNX16 preferentially binds to PI3P. This binding is required for the endosome association of SNX16 since inhibition of PI3P synthesis by wortmannin, an inhibitor of PI3-kinase, results in the diffused distribution of SNX16 in the cytosol of COS-7 cells. The intracellular localization of SNX16 has been investigated in several cell lines; however, the exact distribution pattern of SNX16 appears to be cell type dependent. It has been attributed to EEA1-positive (early endosomes), TFR-positive (recycling endosomes) or Rab7- and Lamp1-positive (late endosomes/lysosomes) dependent on the cell lines used. We demonstrate here that SNX16 vesicles are aggregated near focal adhesions at cell cortex in a variety of cell lines as well as *in vivo*. We propose that these vesicles are early endosomes since they are Rab5-positive. The cell cortex distribution of SNX16 is disrupted upon wortmannin treatment thus it is PI3-kinase dependent, which is consistent with the previous biochemical studies.

SNX23/KIF16B is another PX domain protein and it contains a kinesin domain which is usually involved in the microtubule filament-dependent transport of cargos. Indeed, it has been demonstrated that SNX23 is able to regulate the microtubule-dependent transport of FGFR-containing vesicles or early endosomes. We found that a fraction of SNX23 co-localizes with SNX16 at cell cortex and this observation suggests that SNX23 could be involved in the transport of SNX16 to cell cortex. We performed loss-of-function studies and revealed that SNX23 as well as the microtubule filaments are both required for the cell cortex transport of SNX16. It is interesting to note that SNX16 does not localize to the LBPA-containing multivesicular late endosomes in control Hela cells, however, it re-distributes to this endosomes after the inhibition of microtubule [32]. These observations suggest that a SNX23/microtubule dependent transport route is critical for establishing proper subcellular distribution of SNX16. We tried but failed to detect a direct association between SNX16 and SNX23. It is possible that other adaptor proteins are needed for the SNX23-mediated transport of SNX16.

We report here that SNX16 plays a negative role during the migration or tumorigenesis of MCF-7 cells, but it is dispensable for the growth of these cells. SNX16 mediated vesicular trafficking is involved in signaling pathways such as EGF, BMP and Wnt pathways. However, it is currently unknown whether or not these signaling pathways are involved in cell migration or tumorigenesis in MCF-7 cells. Further studies are required to indentify the exact cargos associated with SNX16 during these processes.

## Conclusions

SNX16-containing vesicles are identified near focal adhesions at cell cortex in addition to their cytosolic distribution. The SNX23/microtubule pathway and the PI3-kinase pathway are both required for the cell cortex distribution of SNX16. SNX16 negatively regulates cell migration *in vitro* and tumorigenesis *in vivo*.

## Methods

### Molecular cloning

Molecular cloning was performed according to standard protocols. Human SNX16, SNX2 and Rab5 genes were amplified from cDNA and cloned into the eukaryotic expression vector pCR3.1-uni-tagged with FLAG, GFP-FLAG or N-GFP. SNX23 was purchased from FulenGen. SNX16 and SNX2 were subcloned into the lentivirus vector PlxnB for establishing stable cell lines. All constructs were confirmed by DNA sequencing. Detailed information about these constructs is available upon request.

### Cell culture, transfection and small chemical treatment

MCF-7, Hela, NCI-H460 and Bel7402 were cultured in RPMI 1640/10% FBS at 37°C with 5% CO_2_. HepG2 and 293T were cultured in DMEM/10% FBS and GLC-82 was cultured in DMEM/10% FBS plus 2 mM L-glutamine. HT1080 was cultured in DMEM/10% FBS plus 0.1 mM non-essential amino acids (NEAA). Transfection was performed using the Lipofectamine 2000 reagent (Invitrogen) according to the manufacturer’s procedure. Stable cell lines were generated by infecting the cells twice with viral supernatants prepared from the 293T cells and colonies were established after selection using blasticidin (Invitrogen, 10 μg/ml) for 72 hrs. The following small chemical inhibitors were used in this study in MCF-7 cells: colchicine (Sigma, 50 μg/ml for 30 min), cytochalasin B (Sigma, 5 μg/ml for 60 min), wortmannin (Sigma, 1 μM for 90 min), monensin (Sigma, 0.1 μM for 90 min), rapamycin (Sigma, 1 nM for 90 min), staurosporine (Sigma, 2 μM for 90 min) and okadaic acid (Sigma, 1 μM for 90 min).

### siRNA treatment and real-time RT-PCR

siRNAs to human SNX16 and SNX23 were designed and synthesized by Ribobio. The target sequences are: siSNX16-1 (CTTTAGAAGAGACAAACTA), siSNX16-2 (AGAAGCAACTTCATATAGA), siSNX23-1 (AGACGAAGTCACTTAGAGA) and siSNX23-2 (AAAGACGCCTTCAGGATTT). Transfection of siRNAs was performed using the DharmFECT transfection reagent (Dharmacon) according to the manufacturer’s protocol and the final concentration of siRNAs was 50 nM. The efficiency of siRNA was determined by real-time RT-PCR at 48 or 72 hrs post transfection. Briefly, total RNA was extracted from cells using the Trizol reagent (Invitrogen). cDNAs were prepared from 5 μg of RNA with the ReverTra Ace® Kit (Toyobo). Quantitative PCR was performed using the Premix Ex Taq™ (Takara) and analyzed with CFX96 Touch™ Real-Time PCR Detection System (Bio-Rad). Three independent assays were performed for each sample and data represents mean ± SD. The primers used are: gapdh (GGGCTGCTTTTAACTCTGGT and TGGCAGGTTTTTCTAGACGG), snx16 (AGAGATGTTTCCAGGTTTTCGAC and AGGCAGTTAGCAATGTCCTTG), snx23 (AGCCCAGATTACGTTTCACAAG and ACAGATCCGAGGTATTAAGCCA).

### Immunofluorescence staining

Cells on glass coverslips were fixed in 4% paraformaldehyde/PBS for 30 min, washed with 2 mg/ml glycine/PBS for 5 min and permeabilized in 0.2% Triton X-100/PBS for 15 min. After two brief washes in PBS, cells were blocked in 3% NGS/PBS for 1 hr at RT. Samples were then incubated in primary antibody for 1 hr at RT. After four washes with 1% BSA/0.05% Tween-20/PBS and three washes with PBS, cells were incubated in Alex 488- or 568-conjugated goat anti-mouse or goat anti-rabbit IgG (Molecular Probe, 1:200) secondary antibody for 1 hr. Cells were then washed four times with 1%BSA/0.05% Tween-20/PBS and three times with PBS, counterstained with DAPI (Sigma, 1 μg/ml) for 3 min and mounted. Mouse heart frozen sections (about 4–5 μM) were prepared using freezing microtome. Sections on slides were fixed in ice acetone for 5–10 min, air dried and then washed with PBS for 10min. Immunofluorescence staining on sections were performed as described above. The anti-SNX16 rabbit polyclonal antibody was home-made in our lab and used at the 1:50 dilution. To test the specificity of the antibody, purified human SNX16 protein (5 μg/ml) was used to block the staining. Other primary antibodies used are: mouse anti-Flag (Sigma, 1:100) and rabbit polyclonal anti-Paxillin (Abcam, 1:25). Images were obtained with the Leica SP2 confocal microscope.

### Cell migration assay

Cell migration was assayed with the Cell Motility HCS Reagent Kit (Cellomics). Briefly, blue fluorescent microsphere solution was added to 24-well plate coated with 1% gelatin. The plate was washed twice with the Wash Buffer after 1 hr incubation at 37°C in the dark. Cells were seeded into the plate (2000 cells/well) and monitored every 2 hrs. Images were analyzed using the Image-Pro Plus 5.0 software (Media Cybernetics). Data represents mean ± SD from three independent experiments.

### Growth curve and cell cycle analysis

Cells were seeded into 24-well plate (20,000 cells/well, triplicated) and cultured as described above. Cells were dissociated from the plate and cell number counted every 24 hrs. For cell cycle analysis, cells were fixed in 70% ethanol for 1hr at 4°C after washing in PBS/1% Glucose and pelleted. Cells were then re-suspended in 1ml of propidium iodide (PI) solution (50 μg/ml PI and 60 μg/ml RNase A) and incubated at 37°C for 1hr. Cells were filtered through 40–70 μm mesh and cell cycle profile was analyzed with the FACSCalibur flow cytometer (BD). Data represents mean±SD from three independent experiments.

### Tumor formation assay

The study was conducted in accordance with the guidelines for the Care and Use of Laboratory Animals in Guangzhou Institutes of Biomedicine and Health (GIBH, CAS). Before transplantation, MCF-7 cells stably expressing SNX16, SNX2 or a control vector were re-suspended in cell culture medium and cell number was counted. Six-week old SCID mice (seven mice/group) were inoculated subcutaneously with the MCF-7 cells (2×10^6^ cells/mouse). Tumors were dissected and weighed 27 days post implantation.
